# Advancements in rice disease detection through convolutional neural networks: A comprehensive review

**DOI:** 10.1016/j.heliyon.2024.e33328

**Published:** 2024-06-19

**Authors:** Burak Gülmez

**Affiliations:** a Leiden Institute of Advanced Computer Science, Leiden University, Leiden, the Netherlands; b Mine Apt, Altay Mah. Sehit A. Taner Ekici Sk. No: 6, 06820, Etimesgut, Ankara, Türkiye

**Keywords:** Artificial intelligence in agriculture, Hyperparameter optimization, Data preprocessing, Convolutional neural networks, Rice disease detection

## Abstract

This review paper addresses the critical need for advanced rice disease detection methods by integrating artificial intelligence, specifically convolutional neural networks (CNNs). Rice, being a staple food for a large part of the global population, is susceptible to various diseases that threaten food security and agricultural sustainability. This research is significant as it leverages technological advancements to tackle these challenges effectively. Drawing upon diverse datasets collected across regions including India, Bangladesh, Türkiye, China, and Pakistan, this paper offers a comprehensive analysis of global research efforts in rice disease detection using CNNs. While some rice diseases are universally prevalent, many vary significantly by growing region due to differences in climate, soil conditions, and agricultural practices. The primary objective is to explore the application of AI, particularly CNNs, for precise and early identification of rice diseases. The literature review includes a detailed examination of data sources, datasets, and preprocessing strategies, shedding light on the geographic distribution of data collection and the profiles of contributing researchers. Additionally, the review synthesizes information on various algorithms and models employed in rice disease detection, highlighting their effectiveness in addressing diverse data complexities. The paper thoroughly evaluates hyperparameter optimization techniques and their impact on model performance, emphasizing the importance of fine-tuning for optimal results. Performance metrics such as accuracy, precision, recall, and F1 score are rigorously analyzed to assess model effectiveness. Furthermore, the discussion section critically examines challenges associated with current methodologies, identifies opportunities for improvement, and outlines future research directions at the intersection of machine learning and rice disease detection. This comprehensive review, analyzing a total of 121 papers, underscores the significance of ongoing interdisciplinary research to meet evolving agricultural technology needs and enhance global food security.

## Highlights

• Utilizing artificial intelligence for efficient rice disease identification.
• Exploration of datasets, classes, and imaging nuances in rice disease studies.
• Presenting the future direction, opportunities, and challenges of AI-driven rice agriculture.

## 1 Introduction

Plants play a vital and multifaceted role in maintaining the health and equilibrium of the planet's ecosystems, proving indispensable for life on Earth. Their significance extends across various ecological, environmental, and societal dimensions. Notably, plants are the primary contributors to the Earth's oxygen supply, absorbing carbon dioxide through photosynthesis and releasing vital oxygen essential for the respiration of diverse organisms, including humans. Acting as carbon sinks, plants contribute to climate regulation by absorbing and storing carbon dioxide, mitigating the impact of greenhouse gases and climate change. Additionally, plants foster biodiversity by providing habitats and sustenance for a diverse array of organisms, forming the foundational support for various ecosystems. From serving as a crucial source of food, including staples like rice, wheat, fruits, and vegetables, to offering medicinal resources and supporting soil stabilization, plants play a pivotal role in human survival and well-being. Their influence extends to regulating the water cycle, enhancing landscapes aesthetically, and contributing significantly to the global economy through industries such as forestry, horticulture, and agriculture. Moreover, plants impact climate regulation by influencing temperature and humidity through processes like transpiration. The integral role of plants in the planet's ecological balance underscores their significance in environmental sustainability and emphasizes the need for conservation and responsible stewardship of these invaluable resources [[1], [2], [3], [4]].

Rice holds paramount importance globally, making significant contributions to human nutrition, food security, and economic sustainability. Serving as a dietary staple for over half of the world's population, particularly in Asia, rice plays a crucial role in providing essential calories and nutrition, serving as a primary energy source for billions. Its high caloric efficiency is pivotal, in meeting the energy needs of large populations. Beyond being a major source of carbohydrates, rice contains vital nutrients, with brown rice retaining more due to minimal processing. Its versatility in culinary traditions worldwide, serving as a base for various dishes, contributes to diverse and culturally rich diets. Additionally, rice holds cultural significance, embedded in practices, traditions, and rituals, symbolizing life, fertility, and prosperity. The economic livelihoods of millions depend on rice cultivation, supporting international trade and contributing significantly to economies. As a staple crop, rice substantially contributes to global food security, addressing hunger and ensuring a stable food supply. Adaptability to diverse agroclimatic conditions and the preservation of traditional rice varieties contribute to agricultural biodiversity. The rice industry's impact on global trade and economies, both on small and large scales, underscores its pivotal role in international commerce. Beyond its role as a major food source, rice's cultural significance, economic impact, and contributions to global food security emphasize its versatility, nutritional value, and adaptability in sustaining human life and well-being [[5], [6], [7], [8]].

Rice is one of the most vital crops globally, serving as a staple food for over half of the world's population. Cultivated in diverse regions, rice plays a crucial role in ensuring food security and livelihoods for millions of people, particularly in Asia, where the majority of rice is grown and consumed. According to the Food and Agriculture Organization (FAO) of the United Nations, global rice production reached approximately 500 million metric tons in 2020, with Asia accounting for about 90 % of the total output [9]. China and India are the top rice-producing countries, with China leading at 149.0 million metric tons and India following closely at 118.0 million metric tons [10]. Additionally, Indonesia, Bangladesh, and Pakistan are among the largest rice-producing countries in Asia [11]. Rice is a crucial staple food for over half of the world's population, providing 60–70 % of caloric intake [12]. The demand for rice is continuously increasing, especially in regions like Asia and sub-Saharan Africa, where populations living in poverty heavily rely on rice as a primary food source [13].

Rice diseases assume critical importance due to their far-reaching consequences on global food security, economic sustainability, and the livelihoods of millions. The significance of addressing and comprehending rice diseases is underscored by several key factors. Firstly, as a staple food for over half of the world's population, particularly in Asia, diseases impacting rice crops directly jeopardize the food supply, affecting the availability of a vital dietary component for billions. Secondly, rice cultivation is a major economic activity supporting the livelihoods of millions of farmers, and diseases can result in substantial economic losses, affecting both small-scale and large-scale rice production and trade. This financial impact can be devastating for farmers and communities reliant on rice agriculture. Additionally, given the significant role of rice in international trade, outbreaks of diseases can disrupt global trade, influencing economies and trade balances. Addressing and preventing the spread of rice diseases is imperative for maintaining stable international commerce. Certain rice diseases can also contribute to environmental degradation, affecting soil health and ecosystem balance, making their mitigation crucial for sustainable agriculture. Moreover, the study of rice diseases stimulates research and innovation in agricultural science, driving advancements in crop protection and sustainable agriculture. Smallholder farmers, constituting a substantial portion of the global agricultural workforce, are particularly vulnerable to the effects of rice diseases, necessitating focused efforts for their resilience and economic well-being. Rice diseases can impact the quality and safety of rice grains, posing risks such as reduced nutritional value, contamination, or the presence of harmful substances. Ensuring the health of rice crops is vital for maintaining the quality and safety of the food supply. The collaborative and international nature of research on rice diseases further emphasizes the need for shared efforts in developing effective strategies and sharing best practices. Addressing rice diseases is imperative due to their direct influence on food supply, economic stability, global trade, environmental sustainability, and the welfare of farmers and communities. Effectively tackling these challenges demands a multidisciplinary approach, integrating scientific research, innovative agricultural practices, and international collaboration to ensure the resilience and sustainability of rice cultivation on a global scale [[14], [15], [16]].

The global rice industry is characterized by a diverse landscape of production, trade, and economic significance. The most prominent rice-producing countries, often referred to as the “rice bowl” nations, include China and India, which collectively account for a substantial portion of the world's rice output. Other major rice producers include countries in Southeast Asia, such as Indonesia, Bangladesh, and Vietnam. In terms of rice trade, there are notable disparities between the largest exporting and importing nations. Typically, countries like India, Thailand, and Vietnam are among the leading rice exporters, while key importers often include nations in Africa, the Middle East, and parts of Asia. The economic importance of rice is multifaceted. It plays a central role in the economies of many nations, particularly in Asia, where rice cultivation supports the livelihoods of millions of farmers. Moreover, the rice industry contributes significantly to international trade, impacting global economies and trade balances. The economic importance extends beyond agriculture, influencing food security, employment, and various related industries. Thus, understanding the dynamics of rice production, trade, and their economic implications is crucial for shaping agricultural policies and ensuring the stability of economies worldwide [[17], [18], [19], [20]].

Today, artificial intelligence (AI) has a wide variety of different application areas [[21], [22], [23], [24], [25], [26], [27], [28], [29]]. The integration of AI into agriculture has opened up new avenues for improving crop management, and one notable application is in the detection of rice diseases. AI, particularly machine learning algorithms, has demonstrated effectiveness in analyzing large datasets of images to identify patterns associated with various diseases. In the case of rice disease detection, AI technologies, such as computer vision and deep learning, offer a promising solution. CNNs are a subset of deep learning algorithms designed for image recognition tasks. They are particularly adept at capturing intricate patterns and features within images, making them well-suited for detecting visual symptoms associated with rice diseases. CNNs excel in discerning complex structures within images, enabling accurate and efficient identification of disease-related patterns. Their usage in rice disease detection involves training the network on diverse datasets containing images of healthy and diseased rice plants. Once trained, the CNN can analyze new images, providing rapid and reliable identification of potential diseases. This AI-driven approach not only enhances the speed and accuracy of detection but also contributes to more proactive and targeted disease management strategies in rice cultivation. In addition to CNNs, there are some alternative algorithms such as Support vector machines, decision trees, random forests, and regression models but they are less effective than CNNs [7,30,31].

This paper stands out among literature review papers in the field of AI-enabled rice disease detection by offering a comprehensive analysis of datasets from diverse regions worldwide, synthesizing insights from over 121 papers to provide a nuanced understanding of the geographic distribution of data collection. It goes beyond superficial descriptions of methodologies, delving deeply into data preprocessing strategies, algorithmic models, and performance evaluation metrics. By identifying challenges and proposing opportunities for improvement, it provides valuable insights for future research directions, emphasizing interdisciplinary collaboration and the development of robust AI algorithms. Additionally, this paper adopts a forward-looking perspective, outlining emerging trends and future research directions in the field. It highlights the global participation of researchers, underscoring the importance of international collaboration, and meticulously examines data sources and preprocessing strategies to provide a deeper understanding of research outcomes and methodologies. Overall, its comprehensive analysis, in-depth methodological examination, forward-looking perspective, emphasis on global participation, and meticulous examination of data sources and preprocessing strategies contribute to its novelty and innovation compared to other literature review papers in the field [[32], [33], [34], [35]].

## 2 Literature review

### 2.1 Rice diseases

Rice is vulnerable to a range of diseases caused by fungi, bacteria, viruses, and other pathogens, posing significant challenges to global rice production. Among the prevalent rice diseases is the devastating Blast Disease, impacting all above-ground parts of the plant with lesions on leaves, stems, and grains, resulting in substantial yield losses. Sheath Blight caused by a fungus affects leaves and sheaths, leading to lesions and rot, thereby diminishing grain quality and yield. Bacterial Leaf Blight induces water-soaked lesions and blighting, causing substantial yield losses. Brown Spot and Rice Blast are fungal diseases impacting leaves, panicles, and nodes, affecting grain development. Other diseases include the Rice Yellow Mottle Virus, Rice Grassy Stunt, Rice Ragged Stunt, Bacterial Streak, and the Rice Water Weevil. Disease management strategies incorporate resistant crop varieties, cultural practices, and innovative technologies like artificial intelligence for early detection and proactive control. This multifaceted approach is crucial for safeguarding global rice production against the threats posed by diverse pathogens and pests [36].

Some rice diseases are not universal, as they vary by growing region. Some rice diseases are universal. For instance, sheath rot is a widespread disease affecting rice crops globally [37]. Rice blast disease, caused by Magnaporthe oryzae, is a major challenge worldwide [38]. Bakanae disease caused by Fusarium fujikuroi is observed in most rice-growing regions [39]. Different regions face distinct diseases; for example, in western Uttar Pradesh, India, Bakanae disease is caused by Fusarium moniliforme [40]. Therefore, while rice diseases like sheath rot and blast disease are prevalent worldwide, the specific types and prevalence of diseases vary by region due to factors such as climate, soil conditions, and agricultural practices.

Active ingredients play a pivotal role in both crop protection and enhancement. For example, a study on pesticide residues in organic rice production in Vietnam identified active ingredients like azoxystrobin, propiconazole, and tebuconazole [41]. A study focusing on rice sheath blight control introduced a dual-functionalized pesticide nanocapsule delivery system that contained validamycin and thifluzamide as active ingredients [42]. Rice byproducts have been found to contain health-promoting properties due to the presence of bioactive molecules such as vitamins, minerals, fiber, and phenolic compounds, making them potential ingredients for fortified foods and supplements [43]. The use of active ingredients extends beyond crop protection to include nutritional and medicinal applications. For instance, pigmented rice varieties are rich in antioxidant and physiologically active ingredients compared to white rice, making them valuable for nutraceutical purposes [44]. Rice bran, a by-product of rice processing, contains bioactive ingredients like magnesium, potassium, phosphorus, and B vitamins, which play a role in regulating physiological functions [45]. Red yeast rice ingredients such as monacolin K (lovastatin) and GABA are known for their health benefits in reducing the risk of circulatory diseases [46]. The availability of active ingredients to rice farmers in various countries is extensive and encompasses a wide array of compounds used for crop protection, nutritional enhancement, and medicinal purposes [47].

The main pathogen threatening the rice crop is Magnaporthe oryzae, which is the causal agent of blast disease. This invasive fungus can infect different parts of rice plants, leading to leaf blast, stem blast, panicle blast, and grain blast. The devastating impact of this pathogen on rice production underscores the significant losses incurred annually due to its destructive nature [48,49].

Rice resistance breeding has emerged as a crucial strategy in managing rice diseases and enhancing crop resilience in major rice-producing countries. For instance, in China, extensive research and breeding programs have focused on developing varieties resistant to bacterial blight, blast, and sheath blight through the incorporation of resistance genes such as Xa21, Pi9, and qSB-9 (TQ) [50,51]. India has made significant strides by leveraging marker-assisted selection (MAS) to introgress multiple disease resistance genes into popular high-yielding varieties [52,53]. In Bangladesh, the International Rice Research Institute (IRRI) has collaborated with local institutions to release several disease-resistant rice varieties, such as BRRI dhan varieties, tailored to the specific biotic stresses in the region [54]. Similarly, research in the Philippines has led to the development of high-yielding, disease-resistant varieties through both conventional breeding and biotechnological approaches, including gene editing techniques like CRISPR/Cas9 [55]. The development of resistant rice varieties in these countries not only mitigates the impact of diseases but also reduces the dependency on chemical pesticides, contributing to sustainable agricultural practices [56]. This focus on resistance breeding underscores the critical role of genetic research and innovation in ensuring food security and agricultural sustainability in regions heavily reliant on rice cultivation.

It is vital to pursue minimal errors in order to ensure the reliable detection and management of diseases, with respect to acceptable error percentages. Although the acceptable percentage of error may differ based on the specific application and the tolerance levels of stakeholders. It can be said that if the output variables are not too many, normal error rates should be less than 5 %. If the output variables are too many and cover lots of diseases, the error rate can be 10%–15 %. Nevertheless, this threshold may be modified in accordance with the severity of the disease, economic implications, and practical feasibility.

### 2.2 Dataset analysis

Table 1 offers a detailed overview of the datasets utilized in diverse studies focused on rice disease detection and they are the suggested variables of the authors. These datasets, drawn from various papers, contribute significantly to the breadth and depth of the research landscape. Notably, the “Collected by the authors” category underscores the proactive efforts of researchers in assembling extensive datasets, each varying in terms of the number of images and classes represented. Examining specific papers reveals the strategic use of established datasets like PlantVillage and Kaggle Rice Disease, as seen in studies by Refs. [8,57], respectively, underlining the importance of leveraging well-established resources. The classification diversity, spanning from 2 to 10 classes, underscores the nuanced and intricate nature of the various rice diseases studied. Researchers have thoughtfully incorporated datasets such as Kaggle Blast and Rust, UCI Rice Leaf Disease, and Mendeley Rice Data, showcasing a judicious mix of widely recognized platforms and datasets crafted by researchers to cater to specific research objectives. This table serves as a valuable resource for researchers, offering insights into the dataset characteristics, enabling meaningful comparisons, and supporting a deeper understanding of the data landscape in rice disease detection studies.

**Table 1:** Variable characteristics of datasets.

Paper	Dataset	Number of images	Number of classes
[57]	PlantVillage	2011	2
[8]	Kaggle Rice Disease	1648	3
[58]	UCI Rice Leaf Disease	2700	3
[59]	Collected by the authors	12000	3
[60]	PlantVillage	8000	2
[61]	Kaggle Rice Disease	12229	4
[62]	Kaggle Blast and Rust	3000	2
[63]	Kaggle Rice Leaf Image	4000	3
[64]	Collected by the authors	2500	5
[65]	PlantVillage	90000	6
[30]	Rice Leaf Disease Dataset	900	9
[66]	Kaggle and UCI datasets	3696	3
[67]	Rice Disease Dataset	500	4
[68]	Kaggle Rice Leaf Disease	10000	4
[31]	Collected by the authors	10083	5
[69]	Collected by the authors	5932	3
[70]	Collected by the authors	5932	4
[71]	Collected by the authors	5932	4
[72]	PlantVillage	4955	4
[73]	Collected by the authors	8911	5
[74]	Kaggle Rice Disease	3355	4
[75]	Mendeley Rice Data	5932	4
[76]	Collected by the authors	1000	2
[19]	Collected by the authors	15210	10
[77]	Collected by the authors	626	4
[78]	Rice Disease Dataset	400	4
[79]	Kaggle Rice Nutrient Deficiency	1156	3
[80]	Collected by the authors	5932	9
[81]	Collected by the authors	7332	4
[82]	Kaggle Rice Disease	1579	3
[83]	PlantVillage	120	3
[84]	Collected by the authors	3416	4
[85]	Mendeley Rice Data	6032	4
[86]	Kaggle Rice Disease	1008	4
[3]	Rice Blast Dataset	2000	2
[87]	Kaggle Rice Disease	1732	3

### 2.3 Data preprocessing strategies

Table 2 meticulously details the diverse preprocessing strategies employed by researchers in the domain of rice disease detection using CNNs. Each entry showcases the thoughtful considerations and techniques applied to enhance the quality and relevance of the datasets before feeding them into the models. Strategies range from basic adjustments, such as size cutting and angle changes [88] and contrast, brightness, and color adjustments [8], to more advanced techniques like principal component analysis (PCA) [89] and dual-tree complex wavelet transform (DTCWT) [90]. Notable is the variety of approaches employed for image enhancement, including the use of Generative Adversarial Networks (GANs) [91], Progressive training, PWGAN-GP method, TIDA method, and Test set imbalance adjustment [92], and the application of Hybrid Gaussian-Weiner (HGW) filters for noise removal [93]. Furthermore, some studies incorporate segmentation techniques, such as Otsu segmentation algorithm [82] and Blue channel segmentation [80], to refine the dataset. The breadth of techniques employed illustrates the evolving nature of preprocessing methodologies, with researchers continuously innovating to ensure the robustness and effectiveness of CNN models in the challenging task of rice disease detection.

**Table 2:** Preprocessing strategies.

Paper	Preprocessing
[88]	Size cutting, angle change, vertical symmetrical mirror image processing
[8]	Adjusting contrast, brightness, and color of the images, applying gaussian filtering to remove noise, normalization
[1]	Random rotation, random noise injection, flipping, resizing, foreground extraction
[58]	Thresholding and clustering, color transforming model
[94]	Affine transformations, variation in brightness and contrast, resizing, random crop, jitter, cutout
[95]	Image rescale, image resize
[89]	PCA
[96]	Dct, padding
[97]	Hyperspectral image correction using, selection of region of interest, calculation of spectral reflectance
[98]	Leaf recognition, rotation, scaling, shifting, shearing, flipping
[99]	Rescaling, rotation, image compression, color adjustment, ratio adjustment
[62]	Resizing, normalization, noise reduction
[100]	Flipping, rotating, noise addition, zooming, background removal, pca, cars, spa
[100]	PCA, competitive adaptive reweighted sampling (cars), successive projections algorithm (spa)
[63]	Resizing, background removal, cropping
[91]	GAN
[66]	Image acquisition, image quality check
[93]	Noise removal using Hybrid Gaussian-Weiner (HGW) filter
[101]	One-hot encoding, non-sequenced nucleotide representation
[68]	Rescaling
[102]	Picture cropping, smoothing
[103]	Flipping, cropping, and resizing images
[104]	StyleGAN2-ADA, the variance of the Laplacian filter to discard blurry and poorly generated images
[105]	Converted to HSV color space, Rotating, adjusting width and height, scaling, flipping
[31]	Progressive re-sizing
[90]	Dual-tree complex wavelet transform (DTCWT), PCA, Discrete cosine transform (DCT)
[106]	Background removal, image cropping, and resizing
[107]	Median Filter (MF)
[108]	Cropping, zooming, contrast variation
[109]	Histogram equalization, flipping, skewing, rotation, zoom, shear transformation, noise addition, distortion
[69]	Fuzzy image processing for color to grayscale conversion, FC-CLAHE technique for fuzzy logic contrast enhancement
[70]	Rotations and flipping
[74]	Affine transformation, perspective transformation
[75]	Rotating, shifting, enlarging, flipping, resizing
[110]	Median filtering
[111]	Random rotation, distortion, shear transformation, vertical flip, horizontal flip, skewing, intensity transformation
[112]	Random rotation, horizontal flip, vertical flip, resizing
[19]	Image rescaling, image resizing, rotation, flipping, shearing, random zooming
[113]	Cropping
[114]	SAM-GAN for data augmentation, GAN for data enhancement
[115]	Histogram equalization
[116]	Flip, rotate, randomly cut, random gray scale transformation and random horizontal flip applied
[78]	Otsus thresholding, horizontal alignment, image resizing
[79]	Zoom, rotation, width shift, height shift, horizontal and vertical flips
[80]	Data cleaning, label validation, balancing classes, reshaping images
[117]	CLAHE (Contrast Limited Adaptive Histogram Equalization), image resizing, pixel scaling, zero-to-one scaling
[82]	Otsu segmentation algorithm, blue channel segmentation, median filter, image size reduction
[118]	Normalization, rotation, deblurring, resizing
[83]	Histogram equalization, Image resizing, Image segmentation
[84]	DMD-based attention-driven pre-processing, Image enhancement, Segmentation, DMD for dynamical systems
[119]	Contrast enhancement technique
[20]	Zooming, flipping, brightness adjustment, distortion
[92]	Progressive training, PWGAN-GP method, TIDA method, Test set imbalance adjustment
[120]	Data cleaning, image segmentation
[121]	Image resizing, wiener filter, CLAHE
[86]	Color and brightness adjustment, horizontal and vertical flipping
[122]	Zoom, flip, rotation, shear, cropping
[87]	Image rotation, flips

### 2.4 Data collection zone

Table 3 provides a comprehensive overview of the diverse geographic origins of data sources and collection zones in studies focused on rice disease detection using CNNs. The table showcases the global participation of researchers, contributing datasets from countries such as India, Bangladesh, Türkiye, China, and Pakistan. The data collection spans a multitude of cities and regions, highlighting the wide-ranging efforts to gather representative datasets. For instance, studies like [7,63] concentrate on data collected from India, while [1] specifically lists Kharagpur, Baragarh, and Puri as collection cities. Bangladesh is a significant contributor to this research domain, with studies like [17] focusing on Sylhet, and [30] collecting data from unspecified locations in Bangladesh. China also emerges prominently, with [105,123] conducting data collection in Qiqihar, Meiris district, Daqing, and Daqing, respectively. This diversity in data sources and zones is crucial for the development of robust CNN models, ensuring the models' adaptability to different environmental and agricultural conditions. It also underscores the collaborative and global nature of research in rice disease detection, emphasizing the need for a broad perspective to address the challenges and nuances associated with varied geographic and climatic conditions.

**Table 3:** Data sources and zones.

Paper	Data collected country	Data collected city
[7]	India	
[1]	India	Kharagpur, Baragarh, Puri
[17]	Bangladesh	Sylhet
[99]	Türkiye	Edirne
[63]	India	
[30]	Bangladesh	
[123]	China, India	Qiqihar, Meiris district, Daqing
[103]	India	
[105]	China	Daqing
[31]	India	Uttar Pradesh, Haryana, Assam
[106]	India	
[124]	India	
[109]	Bangladesh	
[69]	India	Kanuru
[70]	India	
[125]	India	
[72]	India	
[126]	India	
[111]	Bangladesh	
[76]	Pakistan	Kashmore
[19]	India	Erode, Perundurai
[113]	China	Chengdu
[127]	China	Anhui province
[116]	China	Erhe Township, Wuchang, Harbin
[128]	Asian countries	
[129]	China	Guangzhou
[81]	India	Orissa, Imphal
[130]	China	
[131]	China	Nanjing

### 2.5 Researcher addresses

Table 4 sheds light on the distribution of research contributions in the field of rice disease detection using CNNs based on the countries of the researchers. China emerges as the leading contributor with 52 papers, showcasing a substantial and consistent commitment to advancing this area of research. Following closely is India, contributing significantly with 36 papers, underlining the substantial research activity in this country. Bangladesh, Saudi Arabia, and Egypt also make notable contributions, with 6, 4, and 3 papers respectively. This distribution highlights the international collaboration and diverse expertise involved in the exploration of CNNs for rice disease detection. The dominance of China and India emphasizes the substantial research infrastructure and interest in these countries, likely driven by the significance of rice as a staple food and the need for advanced technologies to address agricultural challenges. The contributions from Saudi Arabia and Egypt underscore the global reach of research efforts in this domain, showcasing the collaborative nature of the scientific investigation into leveraging AI for crop disease detection.

**Table 4:** Top 5 countries of the researchers.

Country	Number of papers
China	52
India	36
Bangladesh	6
Saudi Arabia	4
Egypt	3

### 2.6 Used algorithms and models

Table 5 provides a comprehensive overview of the diverse range of algorithms employed in recent studies focused on rice disease detection using CNNs. Researchers have strategically utilized various state-of-the-art architectures and methodologies to achieve high accuracy and robust performance in identifying and classifying rice diseases. Noteworthy algorithms include VGG, ResNet, YOLOv3, RestNETV2 101, YOLOv5, Inception-V3, DenseNet, AlexNet, GoogLeNet, Faster R–CNN, MobileNet, NasNet Mobile, SqueezeNet, SURF, HOG, K-nearest neighbors, Support vector machine, and many others. The algorithms are used commonly in the literature [132,133]. The performance metrics accompanying each algorithm, such as accuracy, precision, recall, F1-score, AUC, segmentation accuracy, Dice coefficient, and Jaccard coefficient, showcase the effectiveness and reliability of these models. The utilization of diverse algorithms indicates the continuous exploration and refinement of models for enhancing the precision and robustness of rice disease detection systems. Researchers have not only focused on accuracy but also considered metrics like sensitivity, specificity, precision, and F1-score, showcasing a holistic evaluation of algorithmic performance. This diversity in algorithmic selection and performance evaluation highlights the multidimensional nature of the efforts to combat rice diseases through advanced machine learning techniques.

**Table 5:** Used algorithms.

Paper	Used algorithms	Performance
[88]	VGG, ResNet	Accuracy: 98.64 %
[8]	YOLOv3, RestNETV2 101, YOLOv5	Accuracy: 0.9904, Precision: >0.96, Recall: >0.96, F1-score: >0.96, AUC: 0.9987, Loss rate: 0.0042
[134]	VGG16, VGG19, Inception-V3, ResNet50	Accuracy: 95.3 %
[135]	Attention residual U-Net, U-Net, Attention U-Net	Segmentation accuracy: 94.11 %, Dice coefficient: 0.9626, Jaccard coefficient: 0.6476
[136]	VGG16, DenseNet121, InceptionV3	Accuracy: 99.2 %, Sensitivity: 98.2 %, Specificity: 98.5 %, Precision: 98.4 %, Recall: 98.2 %, F1-score: 98.5 %
[137]	VGG-16, VGG-19, Resnet50, Resnet101	Accuracy: 95 %, Precision: 97.5 %, Type-I error: 2.3 %, Type-II error: 7.7 %
[1]	DenseNet201, Xception, MobileNetV2, ResNet50	Accuracy: 0.9803,
[58]	DenseNet121, Inceptionv3, MobileNetV2, ResNext101, Resnet152V, EfficientNetB7, Xception, AlexNet, GoogLeNet, VGG, Faster R–CNN, MobileNet, NasNet Mobile, SqueezeNet	Accuracy: 97.9 %
[138]	GoogLeNet	Accuracy: 99.58 %
[59]	VGG-16, GoogleNet	Accuracy: 92.24 %
[139]	SURF, HOG, K-nearest neighbors, Support vector machine, ResNet-20	Accuracy: 96.7 %
[140]	AlexNet, GoogLeNet, ResNet50, MobileNetV3, SVM	Recognition rate: 99.69 %
[17]	CNN, InceptionV3, ResNet50, VGG16, VGG19	Accuracy: 92 %
[141]	VGG16, VGG19, ResNet50, ResNet152, ResNet50V2, ResNet152V2, MobileNetV2, DenseNet121, DenseNet201, InceptionV3, Xception	Accuracy: 84.4 %
[142]	ResNet-CBAM, Random Forest, SVM	Accuracy: 97.21 %, Kappa: 96.55
[60]	AlexNet, ResNet 101, Inception V3	Accuracy: 99 %
[61]	CNN-SVM hybrid algorithm, VGG	Accuracy: 97.1 %
[100]	LS-SVM, SAM, PLS-DA, PCA, CARS, SPA, KNN, RF, self-attention 1D-CNN	Accuracy: 99.93 %
[100]	PCA, RF, AdaBoost, KNN, 2D-CNN, 3D-CNN, HybridCNN, 3D-CSAM-2DCNN	Accuracy: 98.93 %
[143]	RDTNet, SVM, HOG, DCNN, XGBoost, VGG19, UNet, Vgg16, Random Forest, YOLOV3, ADSNN-OB, ML-SFFS	Precision: 99.55 %, F1-score: 99.54 %, Accuracy: 99.53 %
[64]	RlpNet, YOLOv3, AlexNet, GoogLeNet, VGG-16, ResNet-34, FSSD, Faster-RCNN, YOLOv4	Recall: 91.84 %, Precision: 92.14 %, F1-score: 91.87 %, Accuracy: 91.84 %, Mean average precision (mAP): 86.72 %, Detection rate (DR): 93.92 %
[144]	Improved YOLOv5s, Improved YOLOv7-tiny	F1-Score: 0.931, mAP (0.5): 0.961, mAP (0.5:0.9): 0.648
[20]	AlexNet, VGG-19, VGG-16, InceptionV3, MobileNet, ResNet-50	AlexNet: Accuracy 89.4 %, ResNet-50: Accuracy 86.1 %
[145]	AlexNet	Accuracy: 94 %, Average percentage rating: 80.89 %
[146]	Inception layer, Residual connection, Depthwise separable convolution	Accuracy: 99.66 %
[147]	SAMResNet, Basic CNN, VGG16	Accuracy: 97 %
[101]	Deep6mAPred, Deep6mA	Accuracy: 0.9556
[148]	InceptionV3, AlexNet	Accuracy: 99.64 %,
[67]	Xception, ResNet50, MobileNet, VGG16, Inception V3, DenseNet121, ViT, SANET	Accuracy: 98.71 %
[149]	VGG16, ResNet101, MobileNet, EfficientNet-B0	Accuracy: 96.43 %
[104]	Faster-RCNN, SSD	FID score: 26.67, KID score: 0.08, Precision: 0.49, Recall: 0.14, mAP: 0.93 for Faster-RCNN, 0.91 for SSD
[150]	Improved Deep Residual Shrinkage Network (ICDRSN), Densenet, Shufflenet, Mobilenet, Resnet	Precision: 98.89 %, Accuracy: 98.65 %, Recall: 98.68 %
[151]	DSGIResNet_AFF, AlexNet, VGG16, ShuffleNetV2, MobileNetV2, MobileNetV3-Small, MobileNetV3-Large	Accuracy: 98.30 %, Sensitivity: 98.23 %, F1-score: 98.24 %, AUC: 99.97 %
[2]	ResNet-50, Modified Red Deer Optimization Algorithm, Deep Learning Convolutional Neural Network (DLCNN)	Accuracy: 99.68 %, F1-score: 99.71 %
[152]	Residual Network (ResNet), VGGNet, Gated Recurrent Units (GRU)	Accuracy: 99 %
[105]	Inception, ResNet	Accuracy: 98.21 %
[106]	CNN-VGG19 model, K-means clustering, Support Vector Machine (SVM), Canny edge detector	Accuracy: 93.0 %, Sensitivity: 89.9 %, Specificity: 94.7 %, Precision: 92.4 %, F1-score: 90.5 %
[153]	MobileNet, ResNet-20, VGGNet-16, GoogLeNet, AlexNet	Accuracy of 90.71 %
[154]	AlexNet	Recognition rate: 99.23 %
[155]	PaddyNet	Accuracy: 98.99 %
[156]	Deep Convolutional Neuro-Fuzzy Method (DCNFM)	Detection/recognition rate: 98.17 %
[157]	Mobile-Atten, MobileNet-V2	Accuracy: 98.48 %
[14]	DenseNet, Inception	Accuracy: 98.63 %
[72]	VGG16	Accuracy: 96.45 %, loss: 0.09
[126]	SR-DCNN	Accuracy: 98.95 %
[158]	CNNIR-OWELM (CNN with Inception-ResNet v2 and Optimal Weighted Extreme Learning Machine)	Sensitivity: 0.905, Specificity: 0.961, Accuracy: 0.942
[159]	DenseNet-121, SE-ResNet-50, ResNeSt-50	Accuracy: 91 %
[160]	Faster R–CNN	Accuracy: 99.25 %
[75]	InceptionV3, InceptionResnetV2, ResNet50, DenseNet201, MobileNet, EfficientNetB3	Accuracy: 100 %,
[161]	AlexNet, M-Net	Accuracy: 71 %
[111]	VGG16, InceptionV3, MobileNet, NasNet Mobile, SqueezeNet	Accuracy: 93.3 %
[162]	VGGNet, Inception module	Accuracy: 92.00 %
[112]	Inception-V3, VGG-16, Alex Net, MobileNet V2, ResNet-18	Accuracy: 96.23 %
[163]	GoogleNet, ResNet-18, SqueezeNet-1.0, DenseNet-121	Accuracy: 95.6 %
[19]	InceptionResNetV2	Accuracy: 95.67 %
[128]	VGG16 model, Resnet50 model, Densenet121 model	Accuracy: 98.75 %
[79]	InceptionV3, InceptionResNetV2, DenseNet121, DenseNet169, DenseNet201	Accuracy: 98.33 %
[164]	YOLOv3, Faster R–CNN, RetinaNet, Mask R–CNN	Accuracy: 95.6 %
[119]	ResNet, VGG, EfficientNet, MobileNet	Accuracy: 99.67 %

### 2.7 Hyperparameter optimization techniques

Table 6 provides a comprehensive overview of the myriad hyperparameter optimization techniques employed in recent studies focused on enhancing the performance of algorithms for rice disease detection. Researchers have explored a diverse range of methodologies to fine-tune the critical parameters that govern the behavior of machine learning models. Noteworthy approaches include traditional methods such as Grid Search, Random Search, and Bayesian Optimization, as highlighted by Ref. [165]. [7] employ Particle Swarm Optimization (PSO), showcasing the utilization of nature-inspired algorithms for hyperparameter tuning [94]. studied Binary Optimization, Particle Swarm Optimization, and Squared Exponential Kernel, revealing a nuanced strategy for optimization. Other innovative techniques include Improved Backtracking Search Algorithm [95], Rider Henry Gas Solubility Optimization (RHGSO) [6], End-to-End Trainable Attention Module [100], and Weight Cooperative Self-Mapping Chaos Optimization Algorithm (WOACW) [166]. [93] introduce the Exhaustiveness and Brownian Motion-related Elephant Herding Optimization (EBM-EHO) algorithm, showcasing unconventional methodologies. The incorporation of Quantum-Inspired Moth Flame Optimizer [167] and Water Wave Optimization (WWO) [115] emphasizes the exploration of nature-inspired algorithms [69]. proposes the IAOF-CNN algorithm, a dedicated approach tailored for hyperparameter optimization in the context of rice disease detection. Collectively, this table illustrates the diversity in hyperparameter optimization strategies, showcasing the continual efforts to innovate and improve the efficiency of algorithms in agriculture through tailored parameter tuning.

**Table 6:** Hyperparameter optimization techniques.

Paper	Hyperparameter optimization techniques
[165]	Grid search, Random search, Bayesian optimization
[7]	Particle Swarm Optimization (PSO)
[94]	Binary Optimization, Particle Swarm Optimization, Squared Exponential Kernel
[59]	Tuning learning rate and number of iterations
[95]	Improved backtracking search algorithm
[168]	Genetic Algorithm, Firefly Algorithm, Cross-validation, Grid search, Random search, Bayesian Optimization, Adaptive technique
[6]	Rider Henry Gas Solubility Optimization (RHGSO)
[62]	Fine-tuning
[100]	End-to-end trainable attention module
[143]	Bayesian optimization
[166]	WOACW (Weight Cooperative Self-Mapping Chaos Optimization Algorithm)
[93]	Exhaustiveness and Brownian Motion-related Elephant Herding Optimization (EBM-EHO) algorithm
[167]	Quantum-Inspired Moth Flame Optimizer
[67]	Bayesian optimization
[107]	OLIHFA-BA
[69]	IAOF-CNN
[71]	Genetic algorithm, Artificial bee colony, Particle swarm optimization
[125]	Grid search
[158]	Flower Pollination Algorithm (FPA)
[111]	Hyperparameter tuning
[169]	Particle swarm optimization, Artificial fish swarm optimization (AFSO), Efficient artificial fish swarm optimization (EAFSO)
[19]	Fine-tuning
[114]	Grid search
[115]	Water Wave Optimization (WWO)
[78]	Bayesian optimization
[170]	Genetic algorithm
[80]	Hyperparameter tuning

## 3 Summary

### 3.1 Challenges

The integration of artificial intelligence into rice disease detection and management faces a spectrum of challenges spanning technological, agricultural, and socioeconomic dimensions. These challenges collectively impact the successful implementation and widespread adoption of AI within the intricate ecosystem of rice farming [146].

Firstly, the development and training of AI models heavily hinge upon the quality and quantity of available datasets. Challenges arise in obtaining diverse and representative datasets for various rice diseases due to factors such as environmental variations, disease prevalence, and data accessibility. One significant challenge is the interpretability of CNNs, often referred to as “black box” models. The inability to explain the decision-making process of CNNs poses a challenge in gaining the trust of stakeholders, particularly farmers and agricultural experts, who may require insights into how predictions are made. CNNs may encounter difficulties in generalizing well to diverse and unseen conditions. Variability in factors such as rice varieties, growth stages, lighting conditions, and environmental factors can affect the performance of CNN models, leading to suboptimal results in real-world agricultural settings [157,171].

Another limitation is the resource-intensive nature of CNN training, which requires significant computational resources and expertise. Smallholder farmers, who constitute a substantial portion of the global rice farming community, may face challenges in accessing these resources, hindering their ability to leverage CNN-based solutions effectively [90,172].

The process of annotating and labeling images in datasets for supervised learning poses a significant hurdle. Accurate annotation of rice disease images requires domain expertise, and inconsistencies or errors in labeling can significantly impact the performance of CNN models, further exacerbating the challenges associated with data quality and quantity [172].

The successful implementation of AI in rice disease detection demands interdisciplinary collaboration between computer scientists, agronomists, plant pathologists, and farmers. Establishing effective communication and understanding the specific needs and challenges faced by each group is pivotal [125].

Deploying AI applications often requires robust infrastructure and high-speed connectivity, which may be lacking in remote or rural agricultural areas. Improving infrastructure and ensuring reliable internet access are essential for the seamless integration of AI technologies [149].

The initial investment and operational costs associated with implementing AI-based solutions pose a potential barrier, especially for resource-limited farmers. Developing cost-effective and scalable AI solutions is imperative to make these technologies accessible to a broader farming community [67].

Ethical concerns related to data privacy, ownership, and potential biases in AI algorithms require careful consideration. Establishing transparent and ethical frameworks for AI deployment in agriculture is essential to build trust among stakeholders [88].

The absence of clear regulatory frameworks for AI applications in agriculture can hinder widespread adoption. Developing regulatory guidelines that ensure the responsible and ethical use of AI technologies is imperative [173].

The dynamic nature of agricultural ecosystems necessitates continuous monitoring and evaluation of AI models' performance. Implementing feedback loops and adaptive strategies is crucial for maintaining the effectiveness of AI-based solutions over time [174].

Addressing these limitations requires concerted efforts from researchers, policymakers, technology developers, and agricultural stakeholders. Collaborative endeavors are needed to develop more interpretable CNN models, improve generalization capabilities, and make AI-based solutions accessible to resource-limited farmers. Moreover, establishing transparent and ethical frameworks for AI deployment in agriculture is essential to build trust among stakeholders and ensure the responsible use of AI technologies in rice disease detection and management [14].

### 3.2 Opportunities

The integration of artificial intelligence in rice disease detection and management offers a myriad of opportunities that span technological advancements, agricultural practices, and socioeconomic benefits. These opportunities hold the potential to significantly enhance the effectiveness and sustainability of rice cultivation, providing a positive outlook for the future of AI in the rice farming ecosystem [71].

AI enables precision agriculture through the provision of real-time data and insights to farmers. Utilizing sensors, drones, and satellite imagery, AI assists in monitoring rice crops, identifying diseases at early stages, and optimizing resource use, thereby enhancing productivity and resource efficiency [146].

AI-powered models have the capability to analyze vast datasets for the detection of subtle signs of diseases in rice crops before visible symptoms appear. Early detection facilitates prompt intervention, minimizing the spread of diseases and reducing yield losses, ultimately contributing to improved food security [175].

AI algorithms can tailor crop management strategies based on specific conditions such as soil health, climate, and disease prevalence. This customization optimizes inputs like fertilizers and pesticides, reducing environmental impact and enhancing overall sustainability [67].

The integration of AI fosters the adoption of technology in agriculture, even among smallholder farmers. User-friendly AI applications providing actionable insights empower farmers with valuable information, improving decision-making processes and enhancing overall farm management [90].

The implementation of AI in rice farming opens opportunities for capacity building and training programs. Educating farmers, agronomists, and other stakeholders on AI technologies enhances their understanding, ensuring the successful integration of these tools into agricultural practices [171].

The wealth of data generated by AI applications can inform evidence-based agricultural policies. Governments and policymakers can utilize this data to make informed decisions, allocate resources efficiently, and formulate strategies promoting sustainable agriculture and rural development [88].

The global nature of AI research and development facilitates international collaboration. Collaborative efforts among researchers, institutions, and governments lead to the exchange of knowledge, best practices, and innovative solutions, contributing to collective and accelerated progress in AI applications for rice disease management. The adoption of AI in rice farming opens avenues for economic diversification. Entrepreneurs, startups, and technology companies can explore opportunities in developing and providing AI-based solutions for agriculture, stimulating economic growth and job creation [148].

AI can revolutionize crop breeding by analyzing genetic data to develop disease-resistant varieties with improved yields. This innovation in breeding technologies contributes to crop resilience, reducing dependency on chemical inputs and enhancing long-term sustainability [14].

AI's contribution to the efficiency and resilience of rice cultivation plays a crucial role in addressing the challenges of global food security. Enhanced disease management, increased productivity, and sustainable agricultural practices supported by AI technologies collectively contribute to addressing the needs of a growing world population [173].

Harnessing these opportunities necessitates proactive collaboration among researchers, technology developers, policymakers, and farmers. By capitalizing on the transformative potential of AI in rice disease detection and management, stakeholders can collectively contribute to a more sustainable, resilient, and technologically advanced future for rice agriculture [109].

### 3.3 Future directions

The future directions in the integration of artificial intelligence in rice disease detection and management hold promising avenues for further advancements and improvements in sustainable agriculture. These potential directions encompass technological innovations, research initiatives, and policy considerations, shaping the trajectory of AI applications in the rice farming ecosystem.

Future research should focus on the development of advanced AI models capable of handling complex and dynamic interactions within rice ecosystems. This includes exploring deep learning architectures, reinforcement learning, and ensemble methods to enhance the accuracy and robustness of disease detection models.

Integrating diverse data sources such as satellite imagery, climate data, and soil information can provide a holistic understanding of rice crop health. Future AI systems should be designed to seamlessly integrate multi-modal data, enabling more comprehensive and accurate disease prediction and management.

Implementing edge computing solutions can enhance the real-time capabilities of AI applications in rice fields. Edge devices, equipped with AI algorithms, can process data locally, reducing latency and enabling timely decision-making in remote or resource-constrained agricultural areas.

As AI becomes more integral to decision-making in agriculture, there is a growing need for explainable AI models. Future research should focus on developing models that provide transparent insights into decision processes, ensuring trust and understanding among farmers and stakeholders.

Tailoring AI applications to be user-friendly and accessible to farmers is crucial. Future directions should emphasize the development of farmer-centric AI tools with intuitive interfaces, enabling easy adoption and integration into existing farming practices.

With changing climate patterns, future AI models should account for climate resilience in disease prediction. Understanding the impact of climate change on disease prevalence and developing adaptive AI models will be vital for sustainable rice cultivation.

Establishing collaborative platforms that facilitate knowledge exchange among researchers, farmers, and policymakers is essential. These platforms can accelerate the adoption of AI technologies, share best practices, and foster a collaborative ecosystem for sustainable agriculture.

Governments and international organizations should actively engage in developing clear policy frameworks for the ethical and responsible use of AI in agriculture. This includes addressing data privacy, and ownership, and ensuring fair access to AI technologies for farmers of varying scales.

Future initiatives should prioritize capacity-building programs to enhance the skills and understanding of farmers, agronomists, and extension workers in utilizing AI tools. Training programs can empower stakeholders to effectively implement and leverage AI for improved rice disease management.

Ensuring the long-term sustainability of AI applications in rice farming requires ongoing research on ecological impacts, socio-economic considerations, and scalability. Striking a balance between technological innovation and sustainable agricultural practices is pivotal for the enduring success of AI in rice disease detection and management.

By focusing on these future directions, the field can advance towards a more resilient, inclusive, and sustainable integration of AI in rice agriculture, ultimately contributing to global food security and the well-being of farming communities.

## 4 Conclusion

In conclusion, the integration of AI into rice disease detection marks a significant advancement with far-reaching implications for sustainable agriculture. Recognizing the pivotal role of rice in global ecosystems and human nutrition, addressing challenges in disease management becomes imperative. This review underscores the transformative potential of AI in revolutionizing traditional approaches, offering avenues for early detection, precision agriculture, and tailored crop management strategies. However, it is crucial to acknowledge and tackle challenges such as data quality, algorithm generalization, and resource constraints, particularly for smallholder farmers.

The comprehensive literature review provides insights into existing datasets, preprocessing strategies, and a diverse array of algorithms utilized in rice disease detection. Understanding the global landscape, including the distribution of data sources and researchers, fosters collaborative efforts essential for effective AI implementation. Performance metrics such as accuracy, precision, recall, and F1 score serve as critical benchmarks for evaluating AI model effectiveness.

The summary section sheds light on multifaceted challenges associated with AI implementation, from data quality to ethical considerations. Concurrently, it unveils opportunities such as precision agriculture, economic diversification, and advancements in crop breeding. Future directions emphasize the need for advanced AI models, integration of multi-modal data, and the development of farmer-centric tools, all aimed at fostering a resilient and inclusive integration of AI in rice agriculture.

The synthesis of challenges, opportunities, and future directions outlined in this review provides a roadmap for stakeholders, including researchers, policymakers, and farmers, to collaboratively navigate toward a sustainable and technology-driven future for rice disease detection. Prioritizing capacity building, policy frameworks, and long-term sustainability is essential as we harness the full potential of AI to address global food security challenges and ensure the prosperity of farming communities.

CNN models are particularly effective in the detection of rice diseases because they have the capacity to autonomously extract and learn hierarchical features from images, thereby capturing complex disease patterns. Their resilience to changes in illumination, scale, and rotation renders them suitable for agricultural environments with a variety of environmental conditions. Benefiting from data augmentation and transfer learning, CNNs effectively manage large datasets, thereby improving their accuracy. They can be incorporated with advanced techniques such as attention mechanisms to enhance performance, and their spatial invariance guarantees consistent pattern recognition. In addition, CNNs are capable of processing multispectral and hyperspectral images, which enable the detection of disease symptoms that are not visible to the naked eye. Consequently, they can provide a precise and early identification of rice diseases.

CNNs have greatly increased rice disease detection accuracy and timeliness. These algorithms can accurately detect and categorize numerous illnesses using huge and varied information, which is essential for early intervention and disease management. Machine learning models may be scaled and tailored to diverse locales and situations to meet rice-growing region illness characteristics. Machine learning in rice production will benefit from integration with IoT, drones, and remote sensing, which can give real-time data inputs and improve disease monitoring and management models. Large, annotated datasets and machine learning model interpretability remain problems despite these encouraging advances. To overcome these problems, future research should construct more visible and explainable models and improve data gathering and annotation. Machine learning may also improve farm sustainability. These technologies enable tailored pesticide, fertilizer, and other input usage, reducing environmental impact and improving resource efficiency.

## Data availability statement

No data was used for the research described in the article.

## CRediT authorship contribution statement

**Burak Gülmez:** Writing – review & editing, Writing – original draft, Supervision, Resources, Methodology, Investigation, Conceptualization.

## Declaration of competing interest

The authors declare that they have no known competing financial interests or personal relationships that could have appeared to influence the work reported in this paper.
